# Bystander Intervention in Intimate Partner Violence: A Scoping Review of Experiences and Outcomes

**DOI:** 10.1177/15248380231195886

**Published:** 2023-08-31

**Authors:** Ella Kuskoff, Cameron Parsell

**Affiliations:** 1 The University of Queensland, St Lucia, Australia

**Keywords:** intimate partner violence, bystander intervention, experiences, outcomes, scoping review

## Abstract

Governments across the globe are increasingly implementing policies that encourage bystanders to prevent intimate partner violence (IPV) by intervening in violent or potentially violent situations. While a wealth of research examines the most effective mechanisms for increasing potential bystanders’ feelings of self-efficacy and rates of intervention, there is significantly less evidence demonstrating how effective bystander intervention is at preventing or interrupting IPV. This article thus presents a scoping review of the literature examining the experiences and outcomes of bystander intervention in IPV. Following Preferred Reporting Items for Systematic Reviews and Meta-Analyses, extension for Scoping Reviews guidelines, six databases were searched for relevant peer-reviewed studies published in English between 2001 and 2021. A total of 13 articles were ultimately included in the review. The review highlights that although current knowledge on the topic is highly limited, the combined findings of the studies indicate that immediate responses to bystander intervention are heavily context dependent: victims (and perpetrators) tend to react differently to bystander intervention depending on the type of intervention, the type of violence being used, and their relationship to the bystander. However, we have little to no understanding of the outcomes of bystander intervention, or how these outcomes might vary across different contexts. We argue that a more comprehensive understanding of the immediate and long-term implications of bystander intervention across different contexts is crucial if we are to maximize the effectiveness and minimize the potential for harm resulting from bystander interventions in IPV.

## Introduction

Bystander intervention is becoming increasingly visible as a response to intimate partner violence (IPV). Bystander models foreground the responsibility of community members who witness or are aware of IPV to intervene in the situation and engage strategies to diffuse the violence and/or support victims to remain safe (Powell, 2014). Underpinned by social norms theory, bystander intervention is intended to signal to perpetrators of IPV that their behavior is not acceptable and will not be tolerated, and to signal to other bystanders that taking the initiative to intervene in such situations is morally and socially desirable (Fenton et al., 2019). Over time, and in conjunction with other strategies, theorists posit that such an approach will improve informal social control of IPV, enhance the safety of IPV victims, and ultimately prevent perpetrators’ and potential perpetrators’ use of violence in the future (Gainsbury et al., 2020). In this way, bystander intervention is premised on the assumption that all members of society, not simply perpetrators and criminal justice institutions, have a responsibility to end IPV.

The idea that bystanders have a responsibility to intervene in IPV is becoming increasingly common in policy responses across jurisdictions. Indeed, in recent years, countries such as the United States and Australia have implemented policies that include bystander intervention in IPV as a strategy for violence prevention. For example, in 2017, the United States National Center for Injury Prevention and Control (2017) published *Preventing Intimate Partner Violence Across the Lifespan: A Technical Package of Programs, Policies, and Practices.* Within this document, the creation of protective communities is foregrounded as important for “increas[ing] positive bystander behaviors, which can directly interrupt violence” (p. 19), as well as for “increasing the likelihood that community members will intervene when they witness IPV” (p. 29). Similarly, in current Federal Australian policy, one key measure of success for preventing violence against women is “Increased community-wide intention to intervene when witnessing disrespect and violence against women” (Council of Australian Governments 2022, p. 31). In 2016, the Australian Federal Government committed AUD$3.3 million (approximately USD$2.2 million) for “resources to support bystanders to reinforce positive attitudes where safe to do so” (Australian National Audit Office, 2019).

Reflecting the increasing visibility in policy and public discourse of bystander intervention as a mechanism for addressing IPV, there exists a significant body of literature that examines the approach. Existing reviews demonstrate that this literature focuses, in particular, on evidence for how best to encourage bystanders to intervene in IPV. This includes identifying the factors that facilitate and hinder bystander intervention (e.g., Banyard, 2011; Debnam & Mauer, 2021; Robinson et al., 2022), and evaluating programs designed to educate people (and, in particular, college students) about bystander intervention in IPV (e.g., Storer et al., 2016; Wong et al., 2021). This literature draws heavily on respondents’ knowledge and attitudes about bystander intervention in IPV, as well as respondents’ hypothetical reports of what they *would* do if they witnessed a violent situation.

Although such studies are valuable for measuring changes in knowledge and attitudes, as well as for understanding potential bystanders’ intentions to intervene, they have two key limitations. First, many existing review studies focus on bystander intervention in IPV within school and college campus contexts (e.g., Debnam & Mauer, 2021; Fenton et al., 2015; Robinson et al., 2022; Storer et al., 2016; Wong et al., 2021). Critically, the dynamics of IPV within educational contexts are likely to differ significantly to the dynamics of IPV outside of these contexts. For example, students—and particularly those who live on-campus—are less likely to live with their partners, are less likely to be economically dependent on their partners, and are less likely to have children with their partners compared to older couples or couples who have been together for longer (Shorey et al., 2008). This suggests that within school and college contexts, victims’ lives are less enmeshed with their perpetrators’ lives compared with intimate partners outside of these contexts. This means that the types of violent tactics used by perpetrators and, importantly, the potential avenues for and consequences of bystander intervention, are likely to differ significantly across these contexts.

The second key limitation of existing reviews on bystander intervention is that they provide limited evidence about actual experiences of bystander intervention, or the extent to which such intervention is effective at stopping IPV. The aforementioned policy objectives to improve potential bystanders’ ability to recognize and intervene in IPV, in the absence of evidence regarding how bystander intervention is experienced or the extent to which it leads to positive outcomes for those involved, heightens the risk of encouraging bystanders to intervene in IPV in ways that may be unhelpful—or, indeed, harmful—to victims.

Existing literature forms a strong basis for informing the design and delivery of programs intending to increase potential bystanders’ feelings of self-efficacy and improve overall rates of bystander intervention. While the knowledge produced through these studies is undoubtedly important, there remains a critical gap in the review literature regarding the efficacy of bystander intervention in IPV beyond school and college contexts. An understanding of how victims, perpetrators, and bystanders experience bystander interventions, as well as an understanding of the outcomes of such interventions, is critical to inform the design and delivery of bystander intervention policies and programs moving forward, including identifying whether there is an evidence base to substantiate them.

## Study Aims

This article presents a scoping review of peer-reviewed research that examines the experiences and outcomes of bystander intervention in IPV. Informed by the extent of, and gaps within, the current literature, this review is guided by three key aims: (1) to map the scope of existing international research regarding the experiences and outcomes of bystander intervention in IPV situations; (2) to summarize key research findings regarding how bystander intervention in IPV is experienced by those involved and what outcomes it leads to; and (3) to identify critical gaps in the literature and offer recommendations for future research directions.

Importantly, the goal of this review is not to make specific recommendations to policy. Policy and social contexts differ across countries—and, indeed, within countries—and policy decisions should be made based on rigorous and contextualized evidence. This review seeks to bring together all current evidence on the experiences and outcomes of bystander intervention in IPV so that researchers and policymakers across the globe can more readily identify and understand the gaps in evidence within their relevant contexts.

## Methods

To achieve the above aims, a scoping review methodology was adopted. As Arksey and O’Malley (2005) explain, scoping reviews aim to map existing evidence, particularly in under-researched areas, and highlight critical gaps in need of attention. This positions a scoping review methodology as particularly suited to addressing our aims of mapping, summarizing, and identifying gaps in existing evidence on bystander intervention. Below, we outline in detail our inclusion and exclusion criteria, search protocol, study selection process, and our approach to extracting and analyzing the data. Our review follows the Preferred Reporting Items for Systematic Reviews and Meta-Analyses, extension for Scoping Reviews (PRISMA-ScR) guidelines.

### Inclusion and Exclusion Criteria

Drawing on existing evidence, we created a list of inclusion and exclusion criteria to ensure any included studies spoke directly to our focus on experiences and outcomes of bystander intervention in IPV. We explain and justify each criterion below, all of which were required to be satisfied for a study to be included in the review.

#### Type of violence

Studies were included if they related to some form of IPV. This included studies using terms such as “domestic violence” or “family violence.” Studies that related to IPV in addition to other forms of interpersonal violence (e.g., IPV and sexual assault) were also included. Studies focusing solely on other forms of interpersonal violence (e.g., sexual assault, child abuse) were excluded, based on current evidence regarding the unique contexts, power relations, and interpersonal dynamics involved in IPV compared to other forms of interpersonal violence (Walby & Towers, 2018).

#### Type of bystander intervention

Studies were included if they involved any form of bystander intervention. For the purpose of this review, bystander intervention was understood to occur when a third party (i.e., not the victim or perpetrator) involved themselves in a situation involving IPV. The third party could be anyone and the intervention could take any form. Importantly, however, studies were only included if the bystander intervened of their own accord. Studies where the individual was asked by a victim to intervene were excluded, as these were understood as responses to help-seeking, rather than bystander intervention. Several systematic reviews in relation to third-party responses to victims’ help-seeking already exist (e.g., Gregory et al., 2017; Sylaska & Edwards, 2014), and suggest that such contexts are fundamentally different in that the victim has expressed a need, desire, or readiness for outside intervention—conditions that may not necessarily be in place when a bystander intervenes without being asked. Studies that involved both bystander intervention and responses to help-seeking were ultimately included in the review, as were studies in which the nature of the intervention was ambiguous.

#### Experiences and/or outcomes

Studies were included if they reported on the experiences and/or outcomes of bystander interventions. These experiences and/or outcomes could be from the perspective of any involved party, including the victim, perpetrator, or intervening bystander. Initially, we intended to only include studies with a strong focus on experiences and/or outcomes; however, the paucity of studies fitting this criterion required us to broaden our scope to include studies with *any* mention of experiences and/or outcomes within their results. Studies were excluded if their results made no mention of experiences or outcomes of bystander intervention.

#### Community context

Studies were included if they related to any community context outside of educational settings. Studies were excluded if they focused solely on educational settings, such as school or college campuses. Studies that related to educational settings in addition to non-educational contexts were included in the review. As discussed more fully above, the exclusion of studies focusing solely on educational settings was informed by current literature indicating that experiences of IPV and bystander intervention are likely to differ significantly between educational and non-educational community contexts, as well as the current dearth of understanding regarding the latter (Banyard et al., 2020).

#### Publication type

Studies were included if they were empirical studies, peer reviewed, and published between 1 January 2001 and 31 December 2021. Studies were excluded if they were purely review studies, editorials, or methodology focused, as we were interested in original empirical findings. To ensure all included studies met a quality baseline, non-peer-reviewed studies were excluded. Studies published outside of our set timeframe were also excluded, with the timeframe based on the most recent 20 full years of research at the time the search was conducted.

#### Accessibility

Finally, studies were included if they were published in English and if we had access to a full-text version. Studies were excluded if they were published in a language other than English. Ultimately, all studies returned through our searches met our criterion for having access to a full-text version.

### Search Protocol

Based on the inclusion and exclusion criteria outlined above, we ran a title, keyword, and abstract search using the following Boolean search phrase: (“intimate partner violence” OR “domestic violence” OR “family violence” OR “dating violence” OR “relationship violence”) AND (bystander* OR “informal support” OR “informal social”) NOT (college OR school OR student). This search phrase intentionally sought to capture a breadth of terms to account for differences across countries and disciplines. In particular, the term “informal social” enabled us to capture studies that drew on concepts such as informal social support and informal social control in ways that reflect common understandings of bystander intervention.

The search was undertaken on 9 July 2022 across the following six databases: ProQuest (including Social Science Database, Sociological Abstracts, and Research Library); EBSCOhost (including Criminal Justice Abstracts, MEDLINE, and CINAHL); PubMed; PsycINFO; Scopus; and Web of Science. The search returned a total of 809 studies. After removing 333 duplicates, 476 studies remained for the first round of selection.

### Study Selection

Following Arksey and O’Malley (2005), our screening processes began by identifying the inclusion and exclusion criteria outlined above. Using Covidence software to help manage the screening process, each of the two authors independently assessed each study abstract for relevance. Studies that clearly met the inclusion criteria were sent for full-text review. Studies that clearly did not meet the inclusion criteria were excluded. Studies that neither clearly met nor failed to meet the inclusion criteria were sent to full-text review. Conflicts were resolved through discussion between the two authors.

**Figure 1.**
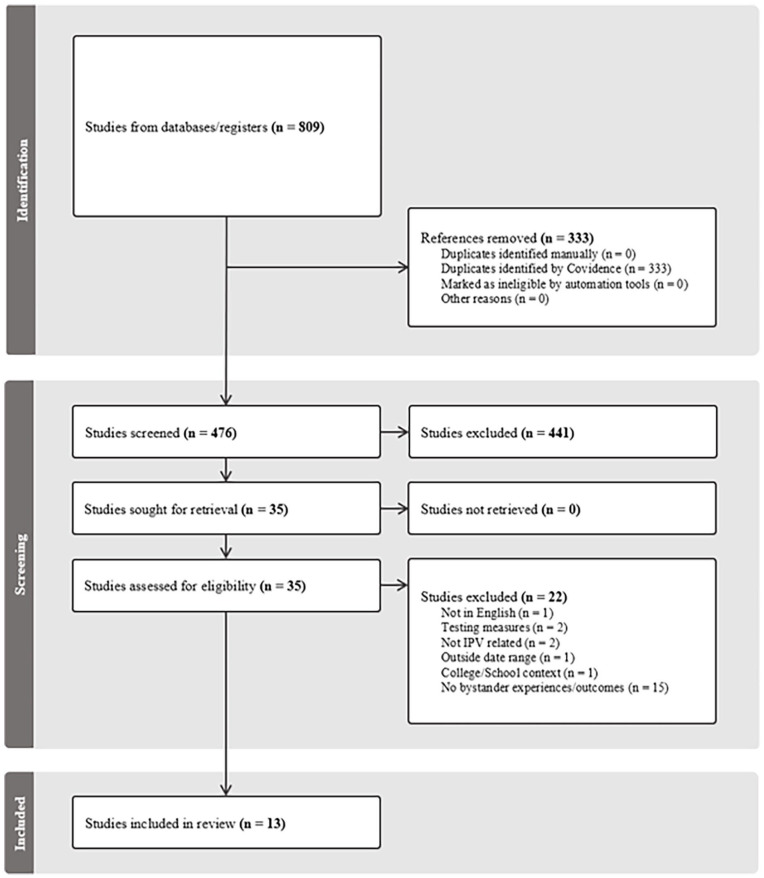
Screening flow diagram.

Through the abstract screen, 441 studies were excluded, and 35 studies were sent for full-text review. The same two authors then independently applied the same inclusion and exclusion criteria for the full-text review. Once again, any conflicts were resolved through discussion between the two authors. This process resulted in a further 22 exclusions, primarily due to no mention of bystander experiences or outcomes (*n* = 15), not being related to IPV (*n* = 2), being methodological papers (*n* = 2), being focused on an educational context (*n* = 1), being in a language other than English (*n* = 1), and being outside the specified date range (*n* = 1). A total of 13 studies were deemed appropriate for inclusion in the scoping review. Figure 1 presents a PRISMA-ScR flow diagram that summarizes this screening process.

### Analysis

Once again drawing on Arksey and O’Malley (2005), we designed a strategy for charting the information in the studies to allow for a basic descriptive analysis. Charting “describes a technique for synthesizing and interpreting qualitative data by sifting, charting, and sorting material according to key issues and themes” (Arksey & O’Malley, 2005, p. 26). Adopting a descriptive-analytical model, the two authors collectively designed a charting framework to guide our collection of the key contextual information from each study. This charting framework included the following: general information (e.g., author, year, study location); conceptualizations (e.g., understandings of bystander interventions, forms of violence); methods (e.g., study design, data collection); participants (e.g., number, age, target group, demographic characteristics); findings reported (e.g., experiences of bystander interventions, outcomes of bystander intervention, type of bystander action taken; perspectives included); and policy implications.

The first author independently charted data from all 13 studies by systematically entering information from each study into an Excel spreadsheet. The second author then randomly selected five of the studies and independently filled in a separate, identical spreadsheet. The information from each spreadsheet was then compared to ensure the accuracy of the information entered. To enable a more in-depth and contextual understanding of the information provided in each study, the studies were also analyzed qualitatively. Using NVivo software, a code was created to correspond with each category included in our charting framework. The first author then coded each study accordingly.

Thus, while the charted information allowed us to provide a basic descriptive analysis to help map the scope and distribution of studies, the qualitative analysis gave us a richer and more contextualized understanding of the studies’ key findings. This enabled us to identify critical gaps in current knowledge surrounding the efficacy and experience of bystander intervention in IPV and recommend areas for future study.

## Results

In this section, we present the results of the scoping review, structured according to the three study aims. These aims were as follows: (1) to map the scope of existing international research regarding the experiences and outcomes of bystander intervention in IPV; (2) to summarize key research findings regarding how bystander intervention in IPV is experienced by those involved and what outcomes it leads to; and (3) to identify critical gaps in the literature and offer recommendations for future research directions.

### Mapping the Scope

In mapping the scope of current knowledge on the experiences and outcomes of bystander intervention in IPV, we were particularly interested in three overarching areas, including (1) the geographic, temporal, and methodological context of the studies; (2) the forms of IPV and bystander intervention considered; and (3) information reported in the findings.

#### Geographic, temporal, and methodological context

Table 1 presents a numerical summary of the geographic, temporal, and methodological contexts of the 13 included studies. The majority of the studies were based in the United States (*n* = 8), followed equally by Australia (*n* = 2), and countries in Asia (*n* = 2). One additional paper presented an international study that included 54 countries. As Table 1 shows, the experiences and outcomes of bystander intervention in IPV are a relatively recent area of research; 53.8% (*n* = 7) of the studies were published between 2019 and 2021.

**Table 1.** Summary: Geographic, Temporal, and Methodological Context.

Region		Year		Study Design		Sample		Sample Size	
	*n* (%)		*n* (%)		*n* (%)		*n* (%)		*n* (%)
USA	8 (61.5)	2020	3 (23.1)	Quantitative	7 (53.8)	Bystanders	4 (30.8)	<50 participants	3 (23.1)
Asia	2 (15.4)	2021	2 (15.4)	Qualitative	4 (30.8)	Women	4 (30.8)	250–400 participants	3 (23.1)
Australia	2 (15.4)	2019	2 (15.4)	Mixed methods	2 (15.4)	Victims	3 (23.1)	600–900 participants	3 (23.1)
International	1 (7.7)	2017	1 (7.7)			Men	3 (23.1)	1,000–2,000 participants	3 (23.1)
		2014	1 (7.7)			General population	1 (7.7)	6,000+ participants	1 (7.7)
		2012	1 (7.7)						
		2011	1 (7.7)						
		2009	1 (7.7)						
		2008	1 (7.7)						

*Notes.* Three of the studies included two groups of participants (e.g., women living in Beijing and women living in Seoul). In these instances, the sample categories and sizes were combined.

The percentages in the “Sample” column add to more than 100% as two studies specifically targeted women victims. These studies are counted both as having “Victims” samples and “Women” samples.

The majority of the studies (*n* = 7) took a quantitative methodological approach, with fewer studies presenting qualitative (*n* = 4) and mixed-methods (*n* = 2) analyses. In the qualitative studies, all data were collected using interviews (*n* = 4). By contrast, all of the quantitative and mixed-methods studies drew their data from surveys (*n* = 9). Studies targeted participants from several core groups, including bystanders with experiences of intervening in IPV (*n* = 4), women (*n* = 4), men (*n* = 3), victims of IPV (*n* = 3), and the general population (*n* = 1). Sample sizes varied greatly, from the smallest sample size (18 participants) to the largest sample size (6,010 participants).

Table 2 provides additional detail on the sample characteristics, including age, sex, ethnicity, and sexuality. Most studies provided information on the age (*n* = 11), sex (*n* = 13), and ethnicity (*n* = 9) of participants. Fewer provided information on participants’ sexuality (*n* = 3). Participant ages ranged from 15 to 91, with reported means ranging from 19 to 45.4 years. Four studies only included female participants, and three studies only included male participants. Most of the remaining studies had a relatively even proportion of female and male participants. Notably, only one study explicitly included participants identifying as having a non-binary or different gender identity. Of the nine studies that provided information on participant ethnicity, eight had a majority of Caucasian/White identifying participants (ranging from 57.4% to 96.3%). Of the three studies that provided information on participant sexuality, two had a majority of heterosexual-identifying participants (ranging from 81.1% to 95.3%), and one had a majority of homosexual/gay-identifying participants (89.2%).

**Table 2.** Summary: Sample Characteristics.

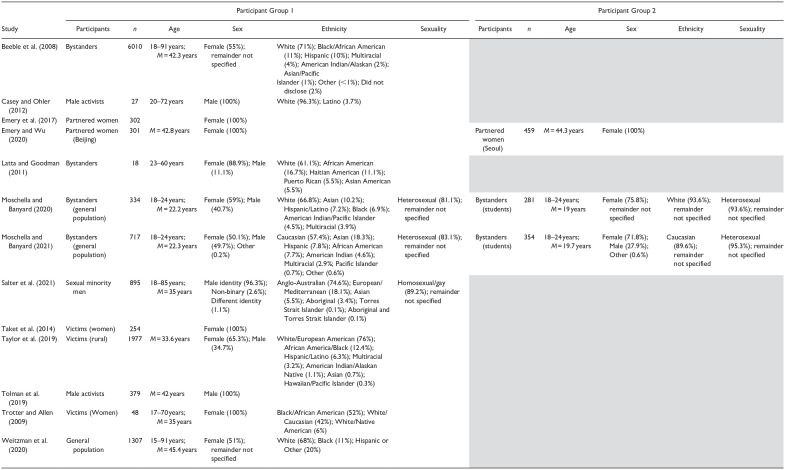

*Notes.* Blank cells indicate that the study did not report relevant information. Shaded cells do not apply to the study.

#### Forms of IPV and bystander intervention considered

Table 3 presents a summary of the different forms of IPV and bystander intervention considered in each of the studies. In most of the studies (*n* = 7), the focus was solely on intimate partner or domestic violence. In a sizeable minority of the studies (*n* = 5), the focus was on intimate partner or domestic violence, as well as sexual violence. The key difference here is that, in contrast to intimate partner or domestic violence where the perpetrator is generally a partner or ex-partner, sexual violence can be perpetrated by anyone. One additional paper took an even broader approach, focusing on gender-based violence (*n* = 1).

**Table 3.** Summary: Forms of IPV and Bystander Intervention Considered.

Type of Violence		Bystander Conceptualization		Bystander Relationship	
	*n* (%)		*n* (%)		*n* (%)
Intimate partner/domestic violence	7 (53.8)	Bystander intervention	7 (53.8)	Friend/family	7 (53.8)
Intimate partner/domestic violence and sexual violence	5 (38.5)	Help-giving	2 (15.4)	Stranger or friend/family	3 (23.1)
Gender-based violence	1 (7.7)	Informal social control	2 (15.4)	Not specified	3 (23.1)
		Informal social support	2 (15.4)		

Reflecting the breadth of our keyword search (and disciplinary conventions), there was some variation in how the studies conceptualized or labeled bystander intervention. The majority of the studies (*n* = 7) drew on notions of bystander interventions. Bystander intervention was not always clearly defined in these studies but generally appeared to refer to third parties (i.e., not the victim or perpetrator) who witnessed and responded to IPV in some manner. The remaining studies equally drew on the concepts of help-giving (*n* = 2), informal social control (*n* = 2), and informal social support (*n* = 2). As noted in the inclusion criteria above, studies that explicitly examined bystander intervention in response to victim help-seeking were excluded, while studies that examined bystander intervention initiated by the bystander were included. Studies where the help-seeking status was ambiguous were also included. A majority of the studies (*n* = 9) focused specifically on forms of bystander intervention that were initiated by the bystanders themselves. In the remaining studies (*n* = 4), it was not clear whether the bystander intervention had been requested by the victims.

The studies also varied in terms of whom they focused on as bystanders. A small majority (*n* = 7) focused on bystander intervention by friends or family members. A further three studies included bystander intervention by friends or family members, as well as strangers. In the remaining studies (*n* = 3), the relationship of bystanders was not specified. Of the total 10 studies that did specify the relationship of the bystander, only three explicitly linked the relationship with the experience or outcome of the intervention.

#### Information reported in the findings

Table 4 presents a summary of the information presented in the studies’ findings, specifically regarding the experiences reported, the type of bystander intervention taken, how different parties (i.e., bystanders, victims, and perpetrators) reacted to the intervention, and the outcomes of the interventions for different parties. A majority of the studies (*n* = 8) presented the perspectives of bystanders, with the remaining studies presenting the perspectives of victims (*n* = 5). No studies presented the perspectives of perpetrators. Most (*n* = 9) of the studies specified the forms of bystander intervention taken. Fewer studies reported how victims (*n* = 5), perpetrators (*n* = 3), and bystanders themselves (*n* = 3) reacted to the interventions. Perhaps most importantly, the outcome of the intervention for the victims was reported most often (*n* = 5), followed by the outcome of the intervention for the bystander (*n* = 2). No studies reported the outcome for perpetrators.

**Table 4.** Summary: Information Reported in the Findings.

Perspectives Reported		Type of Intervention Reported		Reactions Reported		Outcomes Reported	
	*n* (%)		*n* (%)		*n* (%)		*n* (%)
Bystanders	8 (61.5)	Yes	9 (69.2)	Victims	5 (38.5)	Victims	5 (38.5)
Victims	5 (38.5)	No	4 (30.8)	Bystanders	3 (23.1)	Bystanders	2 (15.4)
Perpetrators	0 (0.0)			Perpetrators	3 (23.1)	Perpetrators	0 (0.0)

*Notes.* The percentages in the “Reactions reported” and “Outcomes reported” columns do not add to 100% as some studies reported the reactions and outcomes of multiple parties, while others reported none.

### Summarizing Findings

Studies varied considerably in terms of the number of findings that were relevant to our areas of interest. Some studies’ entire findings were relevant, while others were limited to a few sentences. In this section, we summarize the findings in terms of (1) the actions bystanders took to intervene in IPV, (2) the immediate reactions of the parties involved, and (3) the outcomes of the intervention. Critical findings are summarized in Table 5.

**Table 5.** Summary of Findings.

Study	Bystander Action	Immediate Reactions	Outcomes
Beeble et al. (2008)	• 50% (*n* = 2,975) of the sample had helped someone who was experiencing IPV. • Listening and talking to the survivor (88%) was the most common bystander intervention, followed by referral to an agency (27%), church (17%), or shelter (3%), and informing a relative (22%) or police (18%). Providing practical support, such as a place to stay (10%) or financial assistance (2%), was far less common. • Practical support was significantly more likely to be offered by people who had experienced IPV themselves.	• Bystanders: Not reported • Victims: Not reported • Perpetrators: Not reported	• Bystanders: Not reported • Victims: Not reported • Perpetrators: Not reported
Casey and Ohler (2012)	• 70% (*n* = 19) of the sample reported intervening in violence or disrespectful behavior some, most, or all of the time. • A range of interventions were reported, including talking to the perpetrator (21%), calling out disrespectful language (58%), and directly intervening in abusive situations (24%). • Some bystanders felt more comfortable confronting friends/family members, whereas others were more comfortable confronting strangers.	• Bystanders: Not reported • Victims: Not reported • Perpetrators: Not reported	• Bystanders: Not reported • Victims: Not reported • Perpetrators: Not reported
Emery et al. (2017)	• 4.5% (*n* = 13) of the sample reported that a family member had intervened in their husband’s IPV through protective control. • Protective control included physically standing between the perpetrator and victim, and trying to calm the perpetrator down.	• Bystanders: Not reported • Victims: Not reported • Perpetrators: Not reported	• Bystanders: Not reported • Victims: Protective control by family members was associated with less severe and less injury-producing IPV. • Perpetrators: Not reported
Emery and Wu (2020)	• 3.29% of the Seoul sample and 2.45% of the Beijing sample reported that a friend had intervened in their husband’s IPV through protective control. • 1.85% of the Seoul sample and 1.36% of the Beijing sample reported that a friend had intervened in their husband’s IPV through punitive control. • Protective control included physically standing between the perpetrator and victim, and trying to calm the perpetrator down. • Punitive control included calling the police, threatening the perpetrator, and using physical violence against the perpetrator.	• Bystanders: Not reported • Victims: Not reported • Perpetrators: Not reported	• Bystanders: Not reported • Victims: Protective control by friends was associated with lower IPV severity, whereas punitive control was associated with higher severity. • Perpetrators: Not reported
Latta and Goodman (2011)	• Bystander intervention took many forms, including telling the victim to leave, looking after children, engaging in strategic planning, involving others, and talking to the victim to provide emotional support. A small number of bystanders reported physically intervening in a violent situation. • Bystanders with past experiences of abuse felt better equipped to support victims. • Bystanders felt a greater sense of understanding of the problem and responsibility to intervene when they had a close relationship with the victim.	• Bystanders: Bystanders often struggled with their interventions, particularly when the wishes of the victim went against what the bystanders felt was in the victim’s best interest. Bystanders reported feeling disappointed and sometimes disengaged altogether when they felt the victim was not properly utilizing the advice/support they were offering. • Victims: Not reported • Perpetrators: One interviewee described a situation where they intervened in a perpetrator’s physical attack. The perpetrator laughed at the bystander and continued the attack. Another interviewee described talking calmly to a perpetrator and being able to convince him to go to counseling.	• Bystanders: Not reported • Victims: Not reported • Perpetrators: Not reported
Moschella and Banyard (2020)	• 100% (*n* = 615) of participants reported intervening in IPV/dating violence or sexual assault. • The most common forms of intervention were directly engaging with the perpetrator (62.3%), directly engaging with the victim (24.7%), distancing the victim or perpetrator from the situation (13.5%), directly engaging with both the victim and the perpetrator (8.8%), and delegating responsibility/involving other parties (7%).	• Bystanders: Compared to all other forms of intervention, bystanders were less likely to feel negative feelings and less likely to feel unsafe when they took direct action toward the victim. They were most likely to feel negative feelings and feelings of unsafety when they took direct action toward the perpetrator. • Victims: Victims were less likely to react positively when bystanders took direct action toward the perpetrator. Victims were more likely to react positively when bystanders took distracted action or direct action toward the victim. • Perpetrators: Direct action toward the victim was associated with fewer positive responses from the perpetrator. Perpetrators were most likely to react positively when bystanders took direct action toward the perpetrator.	• Bystanders: Not reported • Victims: Not reported • Perpetrators: Not reported
Moschella and Banyard (2021)	• 100% (*n* = 966) of participants reported intervening in IPV/dating violence or sexual assault. • Type of intervention was not reported. • Bystanders reported more positive experiences and being more likely to intervene again when helping a close friend compared to a stranger.	• Bystanders: 62.6% (*n* = 605) of bystanders felt confident that their intervention stopped the incident and everyone was okay. • Victims: Victims were more likely to react negatively in instances of bystander intervention in dating violence and controlling behavior compared to harassment and unwanted sexual advances. Victims were more likely to react positively when the bystander was a close friend compared to an acquaintance or a stranger. • Perpetrators: Perpetrators were more likely to react negatively in instances of bystander intervention in dating violence and controlling behavior compared to harassment. Perpetrators were more likely to react positively when the bystander was a close friend compared to an acquaintance or a stranger.	• Bystanders: 35% (*n* = 338) reported receiving positive attention from others because of their intervention. 14.9% (*n* = 144) reported being threatened because of their intervention, and 12.2% (*n* = 118) said that intervening ended up costing them a lot of time. • Victims: Not reported • Perpetrators: Not reported
Salter et al. (2021)	• Of the participants who had witnessed IPV (40%), two-thirds intervened in some way. 40.5% (*n* = 133) intervened verbally, 14% (*n* = 46) intervened physically, and 12.5% (*n* = 41) sought help. 10.7% (*n* = 35) provided “other” forms of help, including calling the police and providing emotional support to the victim. • Participants with past experiences of IPV were more likely to report having a friend who has also experienced IPV and were strongly motivated to intervene.	• Bystanders: Not reported • Victims: Not reported • Perpetrators: Not reported	• Bystanders: Not reported • Victims: Some bystanders reported that after intervening in and stopping a violent incident, the relationship between the perpetrator and victim would soon return to “normal” and the violence would continue. • Perpetrators: Not reported
Taket et al. (2014)	• 31% of participants (*n* = 79) reported receiving some form of help from their family and 44% (*n* = 112) reported receiving help from their friends while experiencing IPV. 1% (*n* = 3) reported receiving help from a boss or colleague. • Some specific bystander interventions were reported, including providing emotional support and (unsolicited) advice.	• Bystanders: Not reported • Victims: Many victims expressed that they did not appreciate direct input/advice from bystanders, as this made them feel pressured to act a certain way. Some also did not appreciate bystanders condemning their abusive partners. 60% reported at least one source of informal support as being helpful. 70% reported experiencing support that they found negative or unhelpful. • Perpetrators: Not reported	• Bystanders: Not reported • Victims: Not reported • Perpetrators: Not reported
Taylor et al. (2019)	• Bystanders witnessed approximately one-third of self-reported IPV instances. • Bystanders to physical forms of IPV were usually family members (40%–45.5%) and friends/acquaintances (37.9%–47.7%). Occasionally, police (5.2%–10.4%) and strangers (6.7%–7.6%) were present. • Bystanders to sexual forms of IPV were usually family members (40.9%), followed by friends/acquaintances (22.7%), strangers (22.7%), and police (13.6%). • Specific action taken not reported.	• Bystanders: Not reported • Victims: On average, victims reported that bystanders were most likely to have no impact on the immediate situation (37.7%–50%). This is followed by bystanders who helped (22.7%–41.3%), bystanders whom both helped and harmed (12.7%–23.1%), and bystanders who harmed (6.2%–13.6%). • Perpetrators: Not reported	• Bystanders: One-fifth of victims reported that bystanders were harmed or threatened in some way. • Victims: Victims who were hit, grabbed, or pushed had significantly higher rates of injury when a bystander was present rather than absent. Victims who were hit, threatened, or sexually assaulted experienced higher rates of routine disruption when a bystander was present. When a bystander was harmed, victims were significantly more likely to experience disruption in their routines. Victims who were pushed or grabbed, hit, or beaten up experienced worse mental health when a bystander was present. Overall, the presence of a bystander either had no significant impact on victim outcomes or made the outcomes worse. Even when bystanders helped in a specific situation, this was not associated with better victim outcomes. • Perpetrators: Not reported
Tolman et al. (2019)	• The most common bystander actions taken included talking to young males about respect for women, referring a victim to resources, calling out disrespectful language, and supporting victims.	• Bystanders: Not reported • Victims: Not reported • Perpetrators: Not reported	• Bystanders: Not reported • Victims: Not reported • Perpetrators: Not reported
Trotter and Allen (2009)	• 62.2% (*n* = 28) of participants reported receiving practical support from friends and family in response to IPV. • Participants also commonly reported experiencing emotional support, including having the bystanders listen to their concerns and validate their feelings.	• Bystanders: Not reported • Victims: While some victims reported finding bystanders’ advice useful (particularly in terms of the provision of resources), others felt bystanders were being critical of their decisions or pressuring them to take certain actions. Practical support was well-received by victims—58% (*n* = 28) of victims experienced bystanders calling the police and facilitating their escape as positive interventions. • Perpetrators: Not reported	• Bystanders: Not reported • Victims: Some victims reported bystanders negatively impacting their outcomes by encouraging them to remain in violent relationships or revealing their location to the perpetrator. • Perpetrators: Not reported
Weitzman et al. (2020)	• 26% (*n* = 334) of participants had intervened in IPV, and 8% (*n* = 106) had intervened in sexual assault. • 60% of participants who had intervened in IPV did so by offering victims a safe haven, while 50% told the perpetrator to stop. 25% of participants intervened by involving authorities. • Types of intervention differed for instances of IPV and instances of sexual assault, with bystanders of sexual assault being much more likely to involve authorities. • Participants were 70% less likely to intervene on behalf of an acquaintance compared to a family member.	• Bystanders: Not reported • Victims: Not reported • Perpetrators: Not reported	• Bystanders: Not reported • Victims: Not reported • Perpetrators: Not reported

IPV = intimate partner violence.

#### Bystander actions

The studies demonstrate that bystander intervention in IPV is not an uncommon occurrence in Western countries. Even in studies where bystanders were not specifically targeted as participants, anywhere between 26% (Weitzman et al., 2020) and 76% (Taket et al., 2014) of participants reported that they had either intervened in IPV or have had someone intervene on their behalf. However, the studies also indicate important cultural differences: in Beijing and Seoul, for example, only 2.45% to 4.5% of participants reported having a friend or family member use protective control measures to intervene in IPV (Emery et al., 2017; Emery & Wu, 2020).

The studies also demonstrate that bystanders draw on a range of intervention strategies. Such strategies were categorized in various ways across the studies but generally included interventions targeted at the perpetrator, interventions targeted at the victim, and interventions that involved a third party, such as the police. Interventions targeted at the perpetrator included talking to the perpetrator, calling out disrespectful language, and physically intervening in abusive situations (Casey & Ohler, 2012; Emery & Wu, 2020; Moschella & Banyard, 2020; Tolman et al., 2019; Weitzman et al., 2020). On the other hand, interventions targeted at victims tended to involve removing the victim from the situation, listening to the victim, providing emotional or practical support, or referring to IPV services (Beeble et al., 2008; Latta & Goodman, 2011; Moschella & Banyard, 2020; Salter et al., 2021; Taket et al. 2014; Trotter & Allen, 2009; Weitzman et al., 2020). The frequency of different forms of intervention varied considerably in each study.

The studies point to three important factors that influence the likelihood and type of intervention that bystanders take. The first of these factors is the relationship of the bystander to the victim or perpetrator. Five of the studies explicitly noted that friends and family members are more likely to intervene in IPV compared to acquaintances or strangers. Reasons for this include the closeness of the relationships and the higher likelihood that friends and family will be aware of and witness IPV. Friends and family are also reported to feel a greater sense of responsibility for intervening. The second factor is the type of violence being perpetrated. As shown in Table 2, 6 of the 13 studies looked beyond IPV, and included bystander intervention in a range of sexual assault, harassment, and disrespectful situations. Three of these studies analyzed the differences in bystander responses across different forms of violence. These studies show that bystanders are less likely to be present for physical IPV compared to sexual and other forms of violence and that bystanders tend to respond differently to different forms of violence. Finally, the third factor is the bystander’s own past experiences with IPV. Three studies explicitly highlight how bystanders with previous experiences of IPV are more likely to intervene and are more likely to provide practical support to victims. This finding is explained by a motivation to “pay it forward” for the support they had been given in the past, as well as their first-hand knowledge of what forms of intervention are most useful.

#### Immediate reactions

Six of the 13 studies reported how bystanders, victims, and/or perpetrators reacted to the intervention at the moment. As shown in Table 3, victim reactions were the most commonly reported (*n* = 5). These studies indicate that victims tend to react differently to bystander intervention depending on the type of intervention, the type of violence, and their relationship to the bystander. For example, drawing on bystander perspectives, Moschella and Banyard (2020) found that victims tended to react more positively when bystanders took direct action toward them, and more negatively when bystanders took direct action toward perpetrators. Moschella and Banyard (2021) found that victims tended to react more positively to bystander intervention when they were experiencing harassment or unwanted sexual advances, and more negatively when they were experiencing partner violence or controlling behavior. The same study also found that victims tended to react more positively when the bystander was a close friend rather than a stranger or an acquaintance.

Three studies drew on victim perspectives of their reactions to bystander interventions. These studies found that although victims sometimes experience bystander intervention as having a positive impact on the immediate situation, it is not uncommon for them to see such interventions as having no impact, or even a negative impact. For example, in their quantitative analysis, Taylor et al. (2019) found that bystanders are most likely to have no impact on the immediate situation. This is followed by bystanders who help, bystanders who both help and harm, and bystanders who harm. Drawing on qualitative analyses, Taket et al. (2014) and Trotter and Allen (2009) show that victims find it particularly harmful when bystanders provide direct advice, pressure the victim to take action, or make critical comments about the situation or the perpetrator.

Three of the studies reported how the bystanders felt about their own interventions. Moschella and Banyard (2021) found that 62.6% of bystanders felt confident that their intervention was able to stop the violent incident and that everyone was unharmed. Moschella and Banyard (2020) found that positive bystander feelings were related to the type of intervention taken. For example, bystanders were less likely to feel negative feelings and feelings of unsafety when they took direct action toward the victim, compared to when they took direct action toward the perpetrator and other forms of intervention. Bystanders were most likely to feel negative feelings when they took direct action toward the perpetrator. Qualitatively, Latta and Goodman (2011) showed that victim responses are also critical for shaping how bystanders feel about their intervention. For example, when bystanders thought that victims were not appropriately acting on their advice or were not making the most of the support they were offering, the bystanders expressed negative feelings about intervening.

Three of the studies also reported the perpetrator’s reaction to the bystander interventions. Similarly to victims and bystanders, perpetrators reacted differently depending on the type of intervention, the type of violence, and their relationship to the bystander. Moschella and Banyard (2021) found that perpetrators were more likely to react negatively to bystander intervention in partner violence and controlling behavior compared to bystander intervention in harassment and unwanted sexual advances. Again similarly to victims, perpetrators were more likely to react positively when the bystander was a close friend compared to an acquaintance or stranger. However, Moschella and Banyard (2020) found that the type of intervention favored by perpetrators is opposite to that favored by victims. Whereas victims react more positively to direct intervention toward victims, perpetrators react more positively to direct intervention toward perpetrators. The inverse is also true, whereby direct action toward the victim aroused more negative reactions in the perpetrator, and direct action toward the perpetrator aroused more negative reactions in the victim.

#### Outcomes

Six of the studies reported the outcomes of the intervention for victims, bystanders, and/or perpetrators. Of these studies, five reported the outcomes for victims. These studies provide some evidence that certain forms of bystander intervention may help stop an IPV incident or reduce the severity of IPV. For example, two studies (Emery et al., 2017; Emery & Wu, 2020) found that protective interventions (e.g., physically standing between the victim and perpetrator, trying to calm the perpetrator) were associated with lower IPV severity and fewer injuries. However, four studies also demonstrate that bystander intervention can negatively impact victim outcomes. For example, Emery & Wu (2020) found that punitive interventions (e.g., threatening the perpetrator, using physical violence against the perpetrator) were associated with higher IPV severity. Taylor et al. (2019) found that victims tended to experience higher rates of injury when a bystander was present, as well as greater routine disruption and worse mental health. Trotter and Allen (2009) similarly found that for some victims, bystander intervention was actively harmful to their long-term outcomes, particularly when bystanders encouraged them to take certain actions or provided information about the victim to the perpetrator. Furthermore, Salter et al. (2021) and Taylor et al. (2019) found that even when bystander intervention helped de-escalate violence in the immediate situation, this did not necessarily translate to improved victim outcomes.

Two of the studies reported outcomes for bystanders. Moschella and Banyard (2021) found that 35% of bystanders reported receiving positive attention from others as a result of their intervention. However, 14.9% reported being threatened because of the intervention, and 12.2% indicated that intervening ended up costing them a lot of time. Taylor et al. (2019) found that one-fifth of victims reported that bystanders who witnessed IPV being perpetrated against them were harmed in some way.

None of the studies reported perpetrator outcomes.

### Identifying the Gaps

Given the small number of studies that address the experiences and outcomes of bystander intervention in IPV, there are several critical gaps that require urgent attention.

First, although the studies in our review had vastly different aims and approaches, together they clearly indicate that when it comes to bystander intervention in IPV, context matters. In particular, the type of intervention taken by the bystander, the type of violence being perpetrated, and the relationship between the bystander and the victim or perpetrator were all linked to the immediate experiences and longer-term outcomes of the parties involved. Cultural context also appeared to be an important factor. Given the small number of studies, however, conclusions on how and why bystander intervention operates differently in different contexts are unable to be drawn. Much more research needs to be conducted into the different types of interventions bystanders are using, which forms of violence they are intervening in, and who is intervening to build a picture of what bystander intervention looks like in practice. In addition, only one of the studies explicitly included IPV in sexuality- and gender-diverse relationships. While a majority of studies provided information on the ethnicities of the participants, these did not often feature in the analyses. Much more research is needed on the experiences and outcomes of bystander interventions in diverse relationships and communities.

Second, there are very few studies that report different parties’ immediate reactions to bystander intervention, or the outcomes of the interventions. Those that do exist report some positive immediate and long-term outcomes but also suggest that bystander intervention may actually be more likely to have no impact, or to have a negative impact on the outcomes of various parties. A more comprehensive understanding of the immediate and long-term implications of bystander intervention is crucial if we are to maximize the effectiveness and minimize the potential for harm resulting from bystander interventions. Future research into the implications of bystander intervention should take account of the contextual differences discussed above to provide a better understanding of which forms of bystander intervention are most effective in different contexts.

Third, as shown in Table 3, just over half of the studies rely on the perspectives of bystanders, with the remaining focusing on the perspectives of victims. Although bystander perspectives are indeed important, we should not rely on bystander reports to understand how victims and perpetrators experience and react to bystander intervention. It is critical that future research foregrounds the voices of victims to understand what forms of bystander intervention they find the most useful and in what circumstances, and what forms of bystander intervention are harmful. It is also important for future research to understand the perspectives of perpetrators. Given that perpetrators are the ones enacting the violence, and are the ones whose behavior bystanders are attempting to stop, it is necessary to understand what forms of bystander interventions are most conducive to getting through to the perpetrator without escalating the situation.

## Discussion

Although the outcomes of bystander interventions remain unclear, this review highlights several important implications that should be considered in the design of future studies (these implications are summarized in Table 6). First, the findings of the review reinforce the importance of studying bystander interventions in IPV separately from other forms of dating or sexual violence. In particular, Moschella and Banyard (2021) show that victims and perpetrators react more negatively to bystander intervention in IPV compared to bystander intervention in sexual assault. Similarly, Taylor et al. (2019) found that bystander intervention led to worse reactions/outcomes in some forms of IPV compared to others. This suggests that studies should not only avoid conflating IPV and other forms of violence in their analyses, but they should also consider how bystander intervention may be experienced differently across different types of IPV. For example, physical forms of IPV—and particularly those that pose an immediate threat to a victim’s safety—will likely require different forms of bystander intervention compared to emotional or financial forms of IPV.

**Table 6.** Summary of Implications.

Implications for research	• Future research should avoid conflating IPV and other forms of violence in their analyses and should consider how bystander intervention may be implemented and experienced differently across different forms of IPV. • Future research should examine how bystander intervention is implemented and experienced across different contexts, including within different communities and diverse relationships. • Future research should clearly delineate between bystander intervention and responses to help-seeking. • Future research should capitalize on a range of existing administrative data sources, such as criminal justice and health services data. • Future research should identify types of bystander interventions that are generally well received by both victims and perpetrators.
Implications for policy	• Policymakers should be aware that there is currently little evidence that bystander intervention helps to stop IPV. • Policymakers should understand that experiences and outcomes of bystander intervention are likely to differ across contexts, and have the potential to cause harm.
Implications for practice	• Bystander intervention programs should encourage bystanders to engage empathically with the victim and identify what (if anything) the victim wants from them. • Bystander intervention programs should account for contextual differences and educate potential bystanders about the potential for harm resulting from their interventions. • Bystander intervention programs should evolve and adapt as evidence in this area continues to develop.

IPV = intimate partner violence

Second, around 30% of the studies did not clearly delineate between bystander intervention and victim help-seeking. The former relies on the bystander to recognize IPV and take the initiative to intervene; the latter relies on the victim to ask the bystander for their help. In instances where a victim specifically requests help, it is likely that the bystander intervention is more likely to be useful as the victim has identified intervention as something that they want or need. A substantial body of literature examines help-seeking and subsequent responses in IPV situations (e.g., Gregory et al., 2017; Sylaska & Edwards, 2014). Given that much less is known about bystander intervention, future research should focus on bystander intervention as separate from responses to victim help-seeking.

Third, current studies rely heavily on surveys and, to a lesser extent, qualitative interviews. There exists a range of other potential, rich sources of data that may help provide a more nuanced and comprehensive picture of bystander intervention in IPV. For example, the statistical analyses of administrative data, such as criminal justice and health services data, offer a wealth of opportunities for examining the impacts and outcomes of bystander intervention.

Fourth, although the evidence on victim and perpetrator responses to bystander intervention is currently limited, Moschella and Banyard (2020) find that victims and perpetrators have opposing preferences for the type of bystander intervention. Perpetrators react more positively to interventions targeted at them and less positively to interventions targeted at victims. Victims, however, react more positively to interventions targeted at them and less positively to interventions targeted at perpetrators. This raises important questions about whether it is more critical to design our responses to be positively received by the victims we are trying to protect, or the perpetrators whose behavior we are trying to change. With further research, we may identify and/or develop types of intervention that are well received by both victims and perpetrators.

Finally, the findings of this review highlight that even when bystander intervention is well received and stops the violence in the immediate instance, it does not necessarily lead to improved victim outcomes. This highlights the importance of considering separately the reaction of the victim and perpetrator at the moment and their long-term outcomes. It also draws attention to the complexity involved in IPV and reminds us that even when a single IPV incident is stopped, myriad structural and personal barriers keep victims attached to perpetrators. Stopping a single violent incident is unlikely to result in long-term perpetrator change. Indeed, it may even have the opposite effect, and motivate the perpetrator to adopt more covert or insidious forms of violence to minimize the risk of further unwanted intervention. It is critical that policy strategies avoid putting too many eggs in the proverbial bystander basket—particularly until there is more evidence that bystander intervention helps more than it harms—and ensure that strong provisions are made at the state level for supporting victims of IPV and helping perpetrators to change their behavior.

The findings of this review offer some caution about continued policy advocacy for bystanders to intervene in IPV. Although the implications of this review would not endorse an ending of advocacy for bystander intervention in IPV, the results strongly highlight the need for evidence about the outcomes of bystander intervention. Until there exists a strong evidence base for the effectiveness of bystander intervention, the findings from this review suggest that potential bystanders should engage empathically with victims and identify what victims want from the bystander. Moreover, reflecting the evidence that bystander intervention is more positively experienced when the bystander is close to the victim (and perpetrator), identifying what a victim wants from a potential bystander will likely be best facilitated among people in a close relationship.

## Limitations

To our knowledge, this is the first scoping review to examine the experiences and outcomes of bystander intervention in IPV. Although this review makes a novel contribution, there are two key limitations to note. First, as a means of ensuring the quality of the studies included in the review, we limited our inclusion criteria to peer-reviewed studies only. As such, it is possible that there exists some gray literature that provides insights into the topic, but which was not included in the review. Second, given that the authors are English speakers, we also limited our inclusion criteria to studies published in English. It is possible that this led to the exclusion of some relevant and quality papers published in other languages and diverse cultural contexts.

## Conclusion

This review foregrounds the dearth of studies that examine the experiences and outcomes of bystander intervention in IPV. Although there is some evidence that bystander intervention can be useful in some circumstances, existing studies also highlight that such intervention can be ineffective and at times directly harmful to both victims and bystanders. Given the increasing focus and unprecedented funding that policies are investing in bystander intervention as a protective mechanism against IPV, it is critical that research continues to examine the implications of such interventions for those involved.
